# The Impact of Specialized Knowledge Search on Enterprise Innovation

**DOI:** 10.3389/fpsyg.2021.725514

**Published:** 2021-10-07

**Authors:** Xianyue Liu, Zhipeng Yao, Caixia Liu, Dali Zhao, Chunpei Lin

**Affiliations:** ^1^Business School of Huaqiao University, Quanzhou, China; ^2^Business Management Research Center, Huaqiao University, Quanzhou, China; ^3^College of Economics and Management, North China University of Technology, Beijing, China

**Keywords:** scientific knowledge search, market knowledge search, supply-chain knowledge search, sustaining innovation, disruptive innovation

## Abstract

Against the backdrop of the fierce competition in the market nowadays, a closed innovation model based on internal knowledge is no longer sufficient to support enterprises in search of high-performance innovation. Instead, corporations are desperately required to search for resources of external knowledge to meet their innovation goals. Existing studies on open innovation in corporate management failed to fully elaborate on the mechanism of how the external knowledge search could impose an impact on sustaining innovation and disruptive innovation. In this study, the external knowledge search was divided into three categories according to the knowledge-based theory, namely, the scientific knowledge search, the market knowledge search, and the supply-chain knowledge search. While taking into account the moderating role of the focus of attention of the manager, we analyzed the statistical results of 485 questionnaires collected from manufacturing enterprises to elaborate on the mechanism of how the specialized knowledge search could impose an impact on sustaining innovation and disruptive innovation. Our research conclusions are expected to enrich existing studies on the factors contributing to corporate innovation, including but not limited to sustaining innovation and disruptive innovation. In addition, the research findings are expected to lay an empirical foundation by summarizing previous theoretical opinions while providing references for subsequent in-depth studies in the meantime. Moreover, the paper has put forward practical management measures and suggestions that could enable enterprises in developing countries to search and effectively transform the external knowledge into innovative outcomes. Last but not least, this study is expected to provide both theoretical and practical guidance for enterprises to further facilitate innovation by means of knowledge search.

## Introduction

In the fast-developing economy, technology innovation has played an increasingly important role in shaping competitiveness and industrial transformation and upgrade. Innovation improves the competitiveness and operational efficiency of a company and boosts driving forces for enterprises (Xie and Wang, 2021). The knowledge-based theory emphasized that knowledge is a primary source of corporation innovation whose essence is the commercialization of knowledge. Firms should particularly stress internal knowledge research and external knowledge acquisition to achieve high-performance innovation. If companies fail to break through the complex market condition with their resources and capabilities, they have to leverage external knowledge search to maintain competitiveness and promote development.

Previous scholars have studied the specific process mechanism involving how external knowledge research promoted enterprise innovation from different perspectives based on the scope of knowledge search (Snihur and Wiklund, 2019; Sun et al., 2020), but those researches diverged and reached contradictory conclusions. Local and remote search research scholars believe that local search access familiar knowledge resources at lower costs, strengthening the connection between old and new knowledge elements. In contrast, remote search mitigated the short-sighted and “familiarity” traps problems caused by overwhelming local knowledge if companies only pay attention to local knowledge and bring new technologies and new knowledge that are not locally available (Rosenkopf and Nerkar, 2001). The study of Katila and Ahuja (2002) found an inverted U-shaped relationship between enterprise search behavior and innovative performance, revealing the negative impact of over-search activities. The literature and findings above demonstrate a complex and diverse relationship between knowledge search and enterprise innovation, which needs in-depth study. Previous literature has studied how the impact of the knowledge search on enterprise innovation varies with the research width and depth, but it ignores the fact that knowledge elements resourced from different sources may be different in nature which would further affect innovation activities in distinct ways. Some research explained and verified the significance of external knowledge search in enterprise innovation from various aspects. However, few studied sustaining innovation and disruptive innovation as the results of the application of external knowledge of enterprises or noticed how external knowledge inflow influences the sustaining innovation and disruptive innovation in companies.

In light of the differentiated features of knowledge sources, the knowledge search can be divided into three categories, namely, the scientific knowledge search, the market knowledge search, and the supply-chain knowledge search, whereas these types of knowledge search could be integrated into a unified framework with sustaining innovation and disruptive innovation. In addition, the theoretical model has incorporated the focus of attention of the manager as the adjustment variable, which helped shed light on how external knowledge search could play a role during the sustaining innovation and disruptive innovation of an enterprise.

In this study, we have adopted a large-scale questionnaire survey as the instrument of data acquisition, which was conducted from July 13, 2019, to August 15, 2020. The samples of the field survey were primarily extracted from enterprises and institutions in Fuzhou, Xiamen, and Quanzhou, among other regions in the southern parts of China. The samples of online surveys primarily included Shanghai, Zhejiang, Hubei, Guangdong, and Jiangsu, among other provinces and cities. Subsequently, we have adopted the structural equation modeling (SEM) approach to verify the conceptual model and the research hypotheses. This study is designed to address the following three issues: (1) How may knowledge search of varying categories impose an impact on sustaining innovation and disruptive innovation from the perspective of differentiated knowledge sources? (2) Will the knowledge searched through the method of the same nature impose varying effects on sustaining innovation and disruptive innovation? (3) What sort of moderating roles does the manager's focus of attention play during innovation?

The research conclusions had verified the significance of the external knowledge search for innovation while providing inspiration for enterprises engaged in the innovation practice. In brief, enterprises should choose appropriate innovation methods according to their resource advantages such as high-tech enterprises usually choose disruptive innovation, while medium and low-end technology enterprises usually carry out sustaining innovation. Besides, enterprises should maintain an open mind to focus on contact with external knowledge subjects such as research institutions and supply chain partners. Through interaction and communication, enterprises can obtain a more accurate grasp of market trends and reduce the risk of innovation. The attention-based theory by Ocasio (1997) shows that the attention resources of the managers are limited, so decision-makers should properly allocate internal and external attention, not only strengthen the internal management of the organization and the management of stakeholders but also pay attention to the needs and industry changes of external customers, to grasp the technological frontier of the current market. Previous studies tend to focus on the issue of “where to search” while paying inadequate attention to the issue of “what to search.” To maximize the effect of innovation, enterprises shall opt for the proper method of external search based on their respective actual conditions. This study centered around the factors of external contingency that could impose an impact on corporate innovation to the level of internal managers. Based on the cognitive level of managers, this study has examined the mechanism of how knowledge search could affect sustaining innovation and disruptive innovation when managers have varying levels of attention allocation, to further cope with the “black box” of knowledge search during corporate innovation.

## Literature Review and Research Hypotheses

### Literature Review

During the procedure of Schumpeterian creative destruction, sustainable entrepreneurship would disrupt (either purposefully or coincidentally) the conventional methods of production, products, market structures, and consumption patterns by replacing them with superior, more sustainable (or substantially less unsustainable) products and services. Technology and knowledge in the market are experiencing rapid changes (Schaltegger et al., 2016). The traditional model of closed innovation may lead enterprises to fall into the “competency traps” (Snihur and Wiklund, 2019). To forestall the risk, both scholars and managers suggest relying on the search for external knowledge to make up for the inadequacy in the existing technology and market knowledge of enterprises, which proves to be a method of overcoming the rigid mindset of “not invented here” and “not sold here” (Henry, 2003). According to the knowledge-based theory, the enterprise is regarded as a system of knowledge processing in which tacit knowledge serves as the source of the core competence of enterprises (Pereira and Bamel, 2021). In an enterprise, the knowledge carried by people is shared by such means as texts and technologies and is integrated to create new knowledge that could bring economic value (Cassiman and Valentini, 2016; Gao et al., 2019). Whether the knowledge is developed by the enterprise itself or acquired externally through technological mergers and acquisitions (M&A), supply chain integration, or alliances, such knowledge may translate into a key source of innovation in an enterprise.

The corporate innovation capable of driving sustainable growth does not take place by chance but has to be created by managers who put them at the core of their business models (Schaltegger et al., 2016), which means entrepreneurship is one of the important factors in the construction of corporate innovation. Entrepreneurial culture and entrepreneurship represent the orientation of a firm toward experimenting with new alternatives or approaches by exploring new resources, innovating, and creating new products (Wei et al., 2012; Dabić et al., 2021; Fis and Cetindamar, 2021). Previous research shows that entrepreneurial culture is generally positively related to performance outcomes (Wei et al., 2012; Li and Lee, 2014), which provides resources for corporate to absorb knowledge from the external environment as well as encourage corporate entrepreneurship. The attention-based theory by Ocasio (1997) indicates that what decision-makers focus on and what they do are dependent on the particular context they are in. Since the internal and external conditions vary from one to another, the individual decision-maker may vary their focus of attention depending on the specific internal or external condition. Such variances would impose an impact on the cognition of the management and enterprise, leading to variances in the innovation performance. In general, sustaining innovation integrates and taps into existing knowledge and information to innovate in a slowly improving manner. Through the method of sustaining innovation, enterprises usually search for existing knowledge and elements to enhance and innovate the existing processes, capabilities, and products (Boso et al., 2013). On the other hand, disruptive innovation is based on brand new knowledge and elements, and thus requires a broader scope of knowledge search and knowledge reserve. The disruptive innovation, as a new product, including new service, refers to the procedure of first gaining a strong foothold in simple markets of application, constantly moving upwards to high-end markets, and eventually replacing existing products in the market (Christensen et al., 2018; Williamson et al., 2020). The products newly introduced to the market do not show favorable results in terms of the key performance attributes emphasized by consumers in the mainstream market. However, these products are able to present specific attributes or a combination of attributes, including prices, emphasized by non-consumers, or key performance attributes for over-service customers (Christensen et al., 2018; Roblek et al., 2021), including but not limited to relative simplicity, cheapness, ease of use, or convenience. Such features could help enterprises draw attention from specific consumers to form the niche market, where enterprises could acquire a living space and enhance the capabilities of products to further create sustainable and competitive advantages. In general, disruptive innovation is regarded as a procedure where institutions are trying and exploring new fields (Kammerlander et al., 2018). Enterprises constantly search for opportunities to transform knowledge into innovative outcomes that can facilitate breakthroughs in processes, capabilities, products, and markets (Snihur and Wiklund, 2019).

As for the impact imposed by the knowledge on the innovation processes, knowledge elements, and resource endowments obtained by the institutions, such impact would vary depending on the methods of knowledge search, so would the innovation behaviors and activities of the enterprises (Sun et al., 2020). In this study, the specialized knowledge search was divided into three categories, namely, the scientific knowledge search, the market knowledge search, and the supply-chain knowledge search. Specifically, scientific knowledge search involves the use of knowledge from cooperative universities, research institutes, and governments, or the knowledge collected from academic conferences or forums attended by the search body (Du et al., 2014; McConnell and Cross, 2019; Shen et al., 2020). Moreover, market knowledge search refers to the acquisition of innovation-related knowledge and information from competitors, other industrial enterprises, management consulting agencies, or research institutes engaged in other sectors (Mention, 2011; D'Attnoma and Leva, 2020). Last but not least, the supply-chain knowledge search is deemed to be the insights that focus on suppliers, conferences, industrial associations, and research institutes engaged in the same sector for innovative knowledge and information (Sofka and Grimpe, 2010; Neutzling et al., 2018). Given the definitions, the relationship between varying methods of knowledge search and modes of innovation of start-ups is a theoretical subject worth exploring, so is the variance and implication of such relationship under the adjustment of the focus of attention of the manager.

### Research Hypotheses

#### Impact Imposed by the Specialized Knowledge Search on the Sustaining Innovation and Disruptive Innovation

The strategy of supply-chain knowledge search helps enterprises gain access to more information about suppliers and customers. The study of Mention (2011) has found that upstream suppliers of the value chain are able to offer more information concerning raw materials, whereas the mainstream customer base in the downstream industry could reflect the most popular demands and preferences of customers as well as the creative thinking on product design. Upon acquisition of supply-driven knowledge, enterprises are able to create innovative products with reliable technologies. Moreover, the supply-chain knowledge search may help enterprises tap into the potential demands of the customers so that they are able to accurately identify niche markets that are undervalued and less developed (Ehls et al., 2020). The transformation of raw materials of the suppliers would also impose an impact on the technological and product innovation of enterprises. The new knowledge acquired by enterprises by means of the supply-chain knowledge search may also lead to technological breakthroughs in the secondary performance of existing products. By searching for knowledge from suppliers, enterprises are able to acquire the specialized knowledge at the upper end of the supply chain, such as knowledge on the production of raw materials, methods of manufacturing new products, and developing cutting-edge technologies, which can help enterprises identify the potential technical issues (Tsai, 2009; Jung and Lee, 2016). Such new knowledge can help enterprises rebuild their original business model and technological base, transform the original path of product innovation, and further optimize their products from the aspect of non-critical performance. In addition, the knowledge obtained from suppliers and customers not only helps lower the risks and costs incurred by the development of new products but also facilitates the improvement of the quality of new products (Belderbos et al., 2004; Han et al., 2020). However, the information of the customers is generally intangible and unstructured. With an excessive focus on customer needs, enterprises tend to be short-sighted in conducting research and development (R&D) and compromising the consistency of long-term strategy (Akoumianakis, 2014). In the meantime, excessive search for knowledge on customers and suppliers would lead to information redundancy, blinding the view of useful knowledge. Therefore, we had put forward the following hypotheses:

H1: There is a positive correlation between supply-chain knowledge search and sustaining innovation.
H2: There is an inverted U-shaped relationship between supply-chain knowledge search and disruptive innovation.

Through the market knowledge search, enterprises are able to acquire knowledge on the demands of the mainstream market and the business backgrounds of customers. Subsequently, enterprises may integrate the knowledge with their internal technologies to catalyze the complementary effect generated by varying properties and to further yield greater returns, support the sustaining innovation with more information, and enhance the primary attributes of products. According to the resource-based perspective, the resource allocation in an enterprise will impose an impact on the performance of the disruptive innovation (Lettice and Thomond, 2008; Snihur and Wiklund, 2019). Enterprises may use intellectual properties licensed or purchased from their competitors as an instrument to alleviate their disadvantages in R&D resources and experience (Gans and Stern, 2003; Kishna et al., 2017). Equipped with such knowledge, enterprises are thus able to take advantage of their in-depth insights into the movements and technologies of their competitors to explore the internal value of knowledge of their peer firms and to lay a solid foundation for disruptive innovation (Lin et al., 2015). On the other side of the coin, if conducted excessively, the market knowledge search may exhaust the rare resources of enterprises, thus limiting the possibility of a large-scale search for new knowledge. To make things worse, enterprises may be encountered with fiercer competition and adverse market changes if they focus excessively on existing resources, which hinders the performance of disruptive innovation (Kesavayuth and Zikos, 2012). Therefore, we had put forward the following hypotheses:

H3: There is a positive correlation between market knowledge search and sustaining innovation.
H4: There is an inverted U-shaped relationship between market knowledge search and disruptive innovation.

According to the knowledge-based theory, enterprises are regarded as the system of knowledge processing, whereas tacit knowledge in enterprises serves as the source of core competence (Pereira and Bamel, 2021). The development of an enterprise is the process of acquiring, storing, and producing more knowledge. Moreover, the capability of innovation and application of knowledge enables enterprises to maintain core competitive advantages in the long run (Shen et al., 2020). Universities and scientific research institutes serve as the institutions of knowledge production, which are able to provide enterprises with new scientific knowledge and applied technical knowledge (Yu et al., 2019). The strategy of scientific knowledge search relies on universities and research institutes. Whereas the R&D teams are able to acquire tacit scientific knowledge and (unpublished) compiled explicit knowledge. Such knowledge enables them to promptly match the demands of mainstream consumers in markets with the key performance attributes of products, to develop sustaining innovative products accordingly. In addition, the scientific knowledge search may help enterprises leap out of the conventional technology zone, enhancing the periphery capabilities of products. Enterprises ought to tap into cutting-edge technologies through the institutions of knowledge production and services. Such institutions are expected to facilitate enterprises to timely and rapidly capture external information, knowledge, and technical know-how about innovation during the cultivation of innovative concepts, R&D, industrial, and commercial applications (Choi et al., 2018). The scientific approach and knowledge acquisition channels help eliminate path dependence and organizational inertia, thus creating better conditions for enterprises to usher in disruptive innovation. Therefore, we had put forward the following hypotheses:

H5: There is a positive correlation between scientific knowledge search and sustaining innovation.
H6: There is a positive correlation between scientific knowledge search and disruptive innovation.

#### Moderating Role of the Attention Focus of the Manager

##### Moderating Role of the Internal Attention Focus of the Manager

Based on the theory on management cognition, the external knowledge search involves active monitoring, evaluation of new knowledge, and assessment of information cognition (Li et al., 2013). The increasing internal focus of attention by managers will impose an impact on the enthusiasm for product innovation and the cultivation of corporate competence in innovative thinking. From the aspect of organizational cognition, researchers hold that enterprises could break the inertial “curse” of corporate management with the help of heterogeneous knowledge acquired externally (Liao et al., 2008). When managers pay more attention to the daily operation, institutional mechanisms, and personnel changes, enterprises would become more capable of maintaining an objective and holistic view of internal resources and external environment, thus facilitating the growth of internal creativity (Doloreux, 2015). The research findings of Han et al. (1998) indicated that converting new technologies, though generated internally, into new products requires the collaboration between internal employees with external teams committed to sustaining technology, to create higher commercial value (Deeksha and Ajai, 2013). In addition, by turning its attention to the internal conditions in the enterprise, the management would slow down the absorption of an enterprise of new knowledge from the external technology/market. Such slowdown is favorable for enterprises to consolidate the integration of existing resources and knowledge, to concentrate their primary resources in developing the mainstream market, and to ensure the supply of resources to the sustaining innovation.

H7: The internal attention focus of the manager positively moderates specialized knowledge search and sustaining innovation. Specifically, a higher internal focus of attention would reinforce the positive correlation between specialized knowledge search and sustaining innovation.

##### Moderating Role of the External Focus of Attention of the Manager

Innovation opportunities and new knowledge are mainly originated from outside the enterprise, where uncertain demands, complicated technologies, and intense competition are decisive to the timing of knowledge search. When the management places a heightened focus of attention on the external environment, enterprises are generally able to develop, implement and even reform the strategies to adapt to the changing environment (Wang et al., 2020). The external focus of attention of the manager is a double-edged sword. Where the decision-makers place a heightened focus of attention externally, their enterprises tend to concentrate on external networks of relationships and resources. In the meantime, constantly sourcing knowledge through the supply-chain networks is highly likely to generate redundant resources, leading to a negative marginal utility of external heterogeneity knowledge (Xue and Zhang, 2018). Moreover, the maintenance of a huge supply-chain network risks exhausting the financial and cognitive resources at the disposition of the enterprise and even suffocating the disruptive innovation.

Greater focus on the external environment will encourage enterprises to search for knowledge from competitors, enterprises engaged in other sectors, management consulting agencies, or research institutes involved in other industries. Following this principle, enterprises may apply the searched knowledge to the development of disruptive technologies or the improvement of processes in a faster and bolder manner, while developing new products through trial and error (Harmancioglu et al., 2010). Given the high probability of failure in innovation, the integration of the external focus of attention and the search for market knowledge may lead to endless and repeated experiments, plunging enterprises into an infinite cycle of failures and explorations. Moreover, the absorption and integration of non-peer knowledge would require a significant investment of capital and time, thus undermining the capabilities of enterprises in efficiently utilizing resources in innovation activities (Garriga et al., 2013).

The innovation knowledge on management obtained by enterprises through the strategy of scientific knowledge search tends to be ideological, theoretical, and standardized in general (Xue and Zhang, 2018). These abstract theories fail to elaborate on the details of operation (Damanpour and Aravind, 2012), making it hard for enterprises to directly apply such knowledge to disruptive innovation. For decision-makers who attempt to allocate greater attention in the external focus of attention of the manager, they attach greater importance to the market, technology, or policy opportunities, leaving inadequate energy to transform abstract theories into specific methodologies that help cope with the issues concerning innovation practices. Consequently, enterprises are encountered with greater difficulty in absorbing such highly invisible knowledge in a short time and higher risks of failure during the disruptive innovation.

H8: The external focus of attention of the manager could negatively moderate the supply-chain knowledge search and the disruptive innovation. Specifically, the inflection point of the inverted U-shaped curve between the supply-chain knowledge search and the disruptive innovation will move to the left or the curve will be steeper when the external focus of attention of the manager becomes higher.
H9: The external focus of attention of the manager could negatively moderate the market knowledge search and the disruptive innovation. Specifically, the inflection point of the inverted U-shaped curve between the market knowledge search and the disruptive innovation will move to the left or the curve will be steeper when the external focus of attention of the manager becomes higher.
H10: The external focus of attention of the manager could negatively moderate the scientific knowledge search and the disruptive innovation. Specifically, the positive correlation between the scientific knowledge search and the disruptive innovation will be weaker when the external focus of attention by the manager becomes higher.

**Figure 1:**
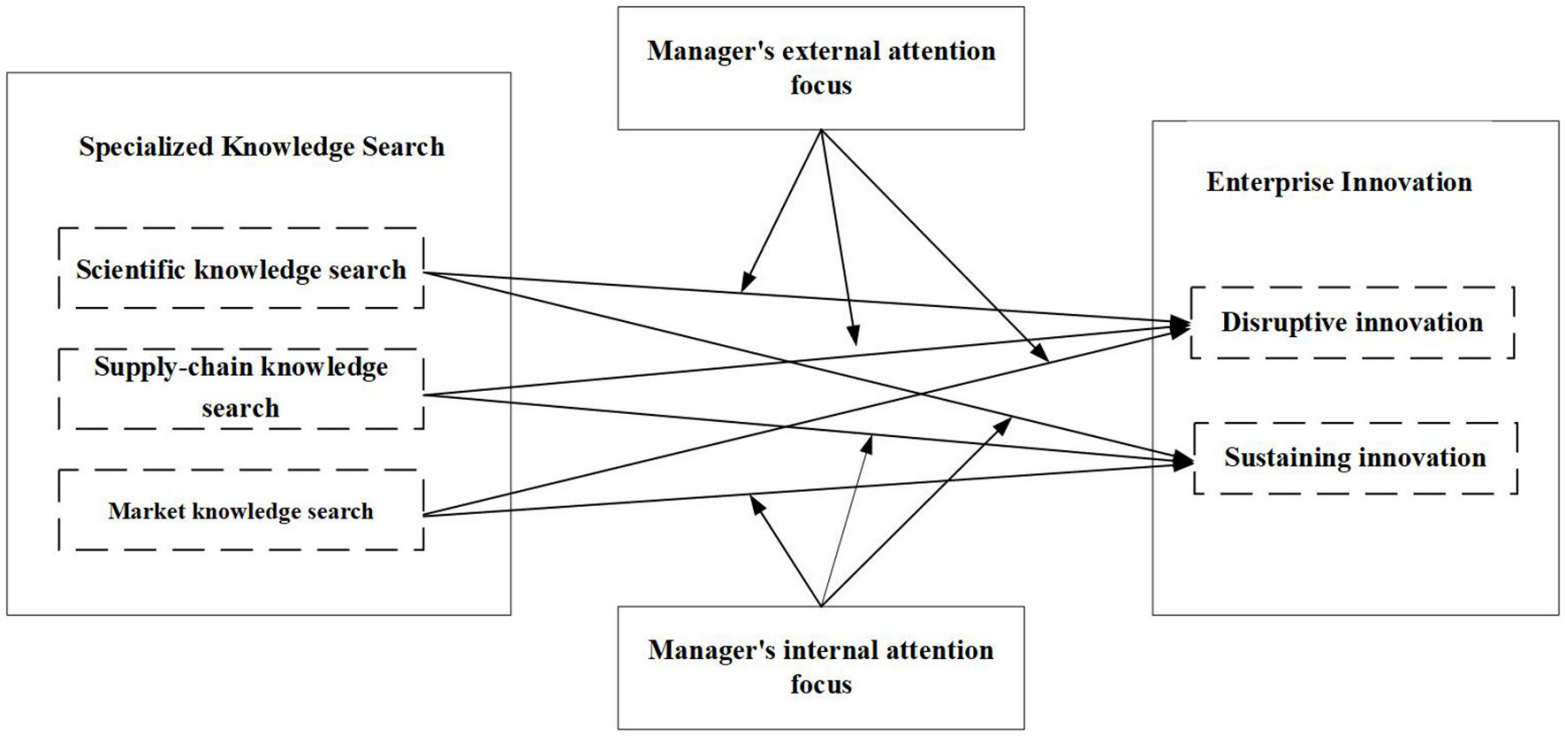
Theoretical model.

The theoretical model based on the aforementioned hypotheses is illustrated in Figure 1.

## The Design of the Research

### Distribution of Questionnaires and Response

In this study, a large-scale questionnaire survey was adopted as the instrument of data acquisition. For the sake of sampling portability, the manufacturers in southern parts of China were selected as the objects of the survey. To enhance the rate of response, the names of enterprises are recorded in the questionnaires, but the individuals who responded were kept anonymous. The respondents selected shall be the management of enterprises with over 1 year of working experience in the same company. Questionnaires were distributed with the assistance of government authorities and industrial associations in good partnerships with the researchers. Specifically, the data were obtained in two steps. First, the survey team issued the questionnaires during on-site investigations at enterprises or to entrepreneurs who attended the seminars organized by government authorities or industrial associations. Second, the survey team contacted the entrepreneurs by following the contact list provided by government authorities or industrial associations and distributed the questionnaires accordingly. The time of formally issuing the questionnaires lasted from July 13, 2019, to August 15, 2020. The questionnaires of on-site surveys were collected from enterprises in Fuzhou, Xiamen, and Quanzhou, among other regions. The questionnaires were filled out on-site by the employees of enterprises/quasi-governmental organizations in Fuzhou, Xiamen, and Quanzhou. In terms of the online survey, questionnaires were distributed on the So-jump to respondents from Fujian, Zhejiang, Shanghai, Hubei, Guangdong, and Jiangsu. A total of 798 questionnaires were collected in total with 485 copies being effective, excluding ineffective questionnaires with omissions (35), questionnaires with the same answer for eight or above consecutive questions (162), and those with contradictory answers for reverse questions (116), representing an effective rate of 60.78%.

### Variable Measurement

#### Specialized Knowledge Search

The measurement toward the specialized knowledge search has drawn inspiration from the research of Sofka and Grimpe (2010), pursuant to which the specialized knowledge search was divided into three categories for measurement, namely, the scientific knowledge search, the market knowledge search, and the supply-chain knowledge search. Questions of measurement included “often obtain innovative knowledge and information in collaboration with colleges and universities, often obtain innovative knowledge and information from competitors, and often obtain innovative knowledge and information from suppliers.” The three sorts of search methods were scored based on the 7-point Likert scale, according to which the Cronbach's α of scientific knowledge search, market knowledge search, and supply-chain knowledge search had the values of 0.849, 0.781, and 0.769, respectively.

#### Attention Focus of the Manager

In this study, the focus of attention of the manager was divided into two dimensions, namely, the internal situation and the external situation. The former dimension intended to measure the attention of the manager to the interests of enterprise organizations, employees, and shareholders, whereas the latter dimension mainly involved the assessment of the focus on the demands of the customers, their purchasing power, and the changes taking place in the industry (Ocasio, 1997; Cho and Hambrick, 2006). The table of scales included six questions, with three questions specified for each of the two dimensions, including “CEO pays much attention to the issues of enterprise organization, CEO pays much attention to the demands of customers, and CEO pays much attention to the changes taken place in the industry.”

#### Sustaining Innovation

In this paper, five questions were designed based on the definition of sustaining innovation with reference to the measurement of the performance of the disruptive innovation. The questions included “The business entities are the pioneers among peers in terms of constantly improving their products in the mainstream market, and the innovation activities in the business entities could satisfy and continue to draw interest from the mainstream market or high-end market.” The sustaining innovation was scored with the Cronbach's α of 0.811 based on the 7-point Likert scale.

#### Disruptive Innovation

During the measurement of the disruptive innovation of an enterprise, the study has drawn inspiration from the experience of most scholars, and the measurement scale developed by Govindarajan and Kopalle (2006) was adopted. Five questions were designed in the study to measure disruptive innovation from the aspects of the degree of destruction, frequency, speed of development, appeal to the market, and process of destruction in relation to the development of products. The questions include “The new products developed by the enterprise could present utterly strong features of disruptive innovation, the enterprise could play a leading role in developing the disruptive products, and the new products developed by the enterprise are utterly attractive to the customers inclined toward anti-establishment and the market-specific customers.” The Cronbach's α of the disruptive innovation amounted to 0.896 based on the 7-point Likert scale.

#### Control Variable

As evidenced by existing studies, the age and size of an enterprise would impose an impact on corporate innovation. In addition, the size of the enterprise would affect the flexibility and adaptability of the organization under specific circumstances. In consistence with these findings, we had taken the age and size of an enterprise as the control variables. The age was measured by the founding years of the enterprise, whereas the size of an enterprise was measured by the total number of its staff. The firm age and firm size were measured by the following items “ <5, 5–10, 10–15 years, more than 15 years; <200 people, 20–300 people, 300–1,000 people, more than 1,000 people.” Given that the research samples originated from varying organizational entities such as group headquarters, subsidiaries, branches, independent corporate enterprises, and business divisions, the varying levels of autonomy of different entities during product innovation would also impose an impact on the overall innovation capabilities of the organization. Therefore, in this research, four additional variables were taken as the control variables, namely, investment in the R&D, product strategic autonomy, industry chain location, and industry attributes, which were assessed by the respondents. In addition, the nature of the property, industry, and enterprise types were taken as the control variables. The details of the questionnaire items are illustrated in Appendix A.

## Data Analysis and Hypothesis Test

### Reliability Analysis

In this study, the reliability of questionnaires was evaluated with Cronbach's α. The results of the reliability test of the sample data are specified in Table 1. According to Table 1, the corrected item-total correlation (CITC) of each measurement question amounted to above 0.475, whereas the Cronbach's α of all the subscales exceeded 0.75, and finally, the Cronbach's α of the scale amounted to 0.933. Judging from the experimental results, all the subscales and the scale featured optimal reliability.

**Table 1:** Reliability test results of subscales and scale.

**Variable**	**Measurement question**	**CITC**	**Cronbach's α**
Disruptive innovation	DI1	0.803	0.896
	DI2	0.821	
	DI3	0.776	
	DI4	0.698	
	DI5	0.633	
Sustaining innovation	SI1	0.568	0.811
	SI2	0.564	
	SI3	0.641	
	SI4	0.664	
	SI5	0.585	
Supply-chain knowledge search	SUS1	0.558	0.769
	SUS2	0.552	
	SUS3	0.545	
	SUS4	0.509	
	SUS5	0.532	
Market knowledge search	MS1	0.603	0.781
	MS2	0.681	
	MS3	0.578	
	MS4	0.495	
Scientific knowledge search	SCS1	0.733	0.849
	SCS2	0.742	
	SCS3	0.719	
	SCS4	0.549	
	SCS5	0.551	
Manager's internal attention focus	IAT1	0.646	0.819
	IAT 2	0.740	
	IAT3	0.640	
Manager's external attention focus	OAT1	0.624	0.818
	OAT2	0.713	
	OAT3	0.676	
The CA of the scale			0.919

### Validity Analysis

#### Confirmatory Factor Analysis

The measurement questions adopted in this study were prepared based on the maturity scale published in authoritative journals. These questions featured optimal content validity subsequent to the improvement through prediction and trial correction. Based on this perception, we conducted confirmatory factor analysis (CFA) to assess the discrimination validity between variables. According to Table 2, the seven-factor benchmark model featured acceptable goodness of fit (χ^2^/df = 2.914, CFI = 0.895, GFI = 0.863, PNFI = 0.749, PGFI = 0.712, RMSEA = 0.063), compared with other alternative models. Judging from the research findings, the discrimination validity of the seven factors in this study was good, indicating that these factors were capable of representing seven distinct constructs.

**Table 2:** Goodness-of-fit index of the measurement model.

**Models**	***χ2***	***DF***	***χ2/DF***	***RMSEA***	***GFI***	***CFI***	***PNFI***	***PGFI***
Seven-factor model	1,119.046	384	2.914	0.063	0.863	0.895	0.749	0.712
Three-factor model	2,616.115	402	6.508	0.107	0.684	0.683	0.598	0.591
Single-factor model	3,718.911	405	9.182	0.13	0.311	0.525	0.464	0.352

*(1) seven-factor model = disruptive innovation, sustaining innovation, supply-chain knowledge search, market knowledge search, scientific knowledge search, manager's internal attention focus, manager's external attention focus; (2) three-factor model = disruptive innovation + sustaining innovation, supply-chain knowledge search + market knowledge search + scientific knowledge search, manager's internal attention focus + manager's external attention focus; (3) single-factor model = disruptive innovation + sustaining innovation + supply-chain knowledge search + market knowledge search + scientific knowledge search + manager's internal attention focus + manager's external attention focus*.

#### Convergent Validity Test

To analyze the convergent validity, we measured the average variance extracted (AVE) of each variable and the coefficient of normalization factor load of each question. As specified in Table 3, the coefficient of normalization factor load of variables to observed variables ranged from 0.622 to 0.928, all of which exceeded 0.5. Except for the AVE of sustaining innovation at 0.399, the AVE of all the other variables exceeded 0.4, indicating that the variables featured optimal convergent validity.

**Table 3:** Convergent validity test.

**Variable**	**Measurement question**	**Standardized load**	***CR***	***AVE***
Disruptive innovation	DI1	0.875	0.896	0.637
	DI2	0.901		
	DI3	0.836		
	DI4	0.710		
	DI5	0.638		
Sustaining innovation	SI1	0.636	0.816	0.472
	SI2	0.651		
	SI3	0.729		
	SI4	0.737		
	SI5	0.675		
Supply-chain knowledge search	SUS1	0.675	0.768	0.399
	SUS2	0.617		
	SUS3	0.620		
	SUS4	0.597		
	SUS5	0.647		
Market knowledge search	MS1	0.682	0.790	0.487
	MS2	0.784		
	MS3	0.690		
	MS4	0.626		
Scientific knowledge search	SCS1	0.798	0.853	0.543
	SCS2	0.847		
	SCS3	0.818		
	SCS4	0.587		
	SCS5	0.589		
Manager's internal attention focus	IAT1	0.737	0.824	0.611
	IAT 2	0.852		
	IAT3	0.751		
Manager's external attention focus	OAT1	0.728	0.821	0.605
	OAT2	0.813		
	OAT3	0.790		

#### Correlation Analysis

Table 4 has specified the mean, standard deviation, and correlation coefficients of variables. Judging from the analytical results, the disruptive innovation was highly correlated with the supply-chain knowledge search (*r* = 0.327, *p* < 0.01), the market knowledge search (*r* = 0.342, *p* < 0.01), and the scientific knowledge search (*r* = 0.301, *p* < 0.01). The sustaining innovation followed a similar pattern given that it has high correlations with the supply-chain knowledge search (*r* = 0.461, *p* < 0.01), the market knowledge search (*r* = 0.528, *p* < 0.01), and the scientific knowledge search (*r* = 0.527, *p* < 0.01). In a nutshell, the results showed that there was a significant correlation among the variables at the significance level of 0.01, thus supporting the hypothesis.

**Table 4:** Pearson correlation coefficient.

	**1**	**2**	**3**	**4**	**5**	**6**	**7**	**8**	**9**	**10**	**11**	**12**	**13**	**14**	**15**	**16**
1. Disruptive innovation	1															
2. Sustaining innovation	0.363***	1														
3. Supply-chain knowledge search	0.327***	0.461***	1													
4. Market knowledge search	0.342***	0.528***	0.585***	1												
5. Scientific knowledge search	0.301***	0.527***	0.477***	0.560***	1											
6. Manager's internal attention focus	0.276***	0.331***	0.334***	0.311***	0.266***	1										
7. Manager's external attention focus	0.326***	0.344***	0.478***	0.421***	0.350***	0.481***	1									
8. Company type	0.118***	0.171***	0.184***	0.158***	0.136***	0.122***	0.165***	1								
9. Company age	0.121***	0.161***	0.152***	0.145***	0.110^**^	0.120***	0.181***	0.246***	1							
10. Number of employees	0.128***	0.227***	0.169***	0.228***	0.280***	0.093^**^	0.178***	0.249***	0.506***	1						
11. Nature of property rights	−0.096^**^	−0.144***	−0.031	−0.072	−0.160***	−0.040	−0.046	0.038	−0.250***	−0.292***	1					
12. Industry	0.129***	0.060	0.160***	0.129***	0.053	0.088*	0.185***	0.069	0.042	0.082*	−0.056	1				
13. R and D investment	0.200***	0.264***	0.138***	0.202***	0.269***	0.224***	0.187***	0.127***	0.234***	0.280***	−0.123***	0.007	1			
14. Product Strategic Autonomy	0.201***	0.249***	0.288***	0.232***	0.217***	0.340***	0.355***	0.156***	0.178***	0.250***	−0.112^**^	0.104^**^	0.548***	1		
15. Industrial chain position	−0.003	−0.070	−0.078*	−0.100^**^	−0.179***	−0.096^**^	−0.051	−0.060	−0.034	−0.065	0.091^**^	−0.014	−0.199***	−0.178***	1	
16. Industry attribute	−0.213***	−0.063	−0.089^**^	−0.150***	−0.123***	−0.157***	−0.092^**^	0.027	0.054	0.015	0.106^**^	−0.079*	−0.119***	−0.184***	0.132***	1
Average value	4.777	5.347	5.597	5.239	5.113	5.039	5.494	2.056	3.068	2.934	2.417	5.392	4.829	5.035	1.732	1.363
Standard deviation	1.232	0.812	0.647	0.787	0.951	1.063	0.806	1.200	0.878	0.769	1.038	3.442	0.861	0.819	0.559	0.481

** *p < 0.01*,

* *p < 0.05*.

### Hypothesis Test

#### Main Effect Test

##### Specialized Knowledge Search and Sustaining Innovation

As specified in Table 5, M1 is the benchmark model, whereas M2, M3, and M4 are derived from M1 through the addition of monomial independent variables. Compared with M1, the *R*^2^ of M2 has experienced significant increases (Δ*R*^2^ = 0.064, Δ*F* = 98.203^***^), indicating that the supply-chain knowledge search featured a significant explanation effect on sustaining innovation. Furthermore, the regression coefficient amounted to 0.521, reaching the significance level of *p* < 0.001. The experimental result could thus support H1.

**Table 5:** Regression results of knowledge search and sustaining innovation.

**Variable**	**Sustaining innovation**			
	**M1**	**M2**	**M3**	**M4**
Constant	3.753^***^	1.498^***^	1.782^***^	2.120^***^
Company type	0.179^*^	0.104	0.112+	0.122+
Company age	0.008	−0.019	−0.001	0.045
Number of employees	0.106+	0.081	0.033	−0.005
Nature of property rights	−0.135+	−0.158^*^	−0.145^*^	−0.075
Industry	0.066	−0.06	−0.045	0.046
R and D investment	0.139^**^	0.159^***^	0.108^*^	0.067
Product strategic autonomy	0.109^*^	0.006	0.059	0.093^*^
Industrial chain position	0.003	0.019	0.029	0.078
Industry attribute	−0.033	0.001	0.061	0.027
Supply-chain knowledge search		0.521^***^		
Market knowledge search			0.492^***^	
Scientific knowledge search				0.407^***^
Adj. *R*^2^	0.107	0.259	0.309	0.301
*F*	7.453^***^	17.900^***^	22.686^***^	21.834^***^
Δ*R*^2^		0.064	0.066	0.051
Δ*F*		98.203^***^	140.137^***^	132.674^***^
Observations	485	485	485	485
VIF	1.03 < VIF <1.73

*** *p < 0.001*,

** *p < 0.01*,

* *p < 0.05*,

*+ p < 0.1*.

Similarly, compared with M1, the *R*^2^ of M3 has experienced significant increases (Δ*R*^2^ =0.066, Δ*F* = 140.137^***^), indicating that the market knowledge search featured a noticeable explanation effect on sustaining innovation. Since the regression coefficient amounted to 0.492, reaching the significance level of *p* < 0.001, the market knowledge search proved to impose a significant positive impact on sustaining innovation. The experimental result could thus support H3.

Finally, compared with M1, the *R*^2^ of M4 has experienced significant increases (Δ*R*^2^ = 0.051, Δ*F* = 132.674^***^), and the regression coefficient of the scientific knowledge search was significantly positive (β = 0.407, *p* < 0.001). Judging from the result, the scientific knowledge search was positively correlated with sustaining innovation, which could support H5.

##### Specialized Knowledge Search and Disruptive Innovation

As specified in Table 6, M5 is the benchmark model, whereas M6, M8, and M10 are derived from M5 with the addition of monomial independent variables, based on which M7, M9 is established with the addition of quadratic independent variables. Compared with M6, the *R*^2^ of M7 was significantly higher (Δ*R*^2^ =0.007, Δ*F* = 5.096^*^), indicating that scientific knowledge search featured a vital explanation effect on disruptive innovation. Moreover, the monomial coefficient of the supply-chain knowledge search was significantly positive (β =0.417, *p* <0.001), whereas the quadratic coefficient of the supply-chain knowledge search was significantly negative (β = −0.166, *p* <0.05). Judging from the result, there was a U-shaped relationship between the scientific knowledge search and the disruptive innovation, which could support H2.

**Table 6:** Regression results of knowledge search and disruptive innovation.

**Variable**	**Disruptive innovation**					
	**M5**	**M6**	**M7**	**M8**	**M9**	**M10**
Constant	3.101^***^	0.868	1.499^*^	1.385^*^	2.116^***^	1.812^**^
Company type	0.192+	0.118	0.119	0.135	0.141	0.147
Company age	0.07	0.044	0.051	0.062	0.077	0.099
Number of employees	0.031	0.006	0.001	−0.033	−0.045	−0.057
Nature of property rights	−0.096	−0.119	−0.124	−0.105	−0.103	−0.049
Industry	0.375^*^	0.251	0.203	0.278+	0.253	0.359^*^
R and D investment	0.170^*^	0.190^*^	0.189^*^	0.143+	0.129+	0.113
Product Strategic Autonomy	0.111	0.008	0.013	0.067	0.079	0.098
Industrial chain position	0.157	0.172+	0.161+	0.179+	0.165+	0.216^*^
Industry attribute	−0.482^***^	−0.449^***^	−0.429^***^	−0.400^***^	−0.393^***^	−0.435^***^
Supply-chain knowledge search		0.516^***^	0.417^***^			
Supply-chain knowledge search **×** Supply-chain knowledge search			−0.166^*^			
Market knowledge search				0.428^***^	0.318^***^	
Market knowledge search **×** Market knowledge search					−0.194^**^	
Scientific knowledge search						0.321^***^
Adj. *R*^2^	0.095	0.159	0.166	0.161	0.176	0.146
*F*	6.668^***^	10.145^***^	9.766^***^	10.267^***^	10.376^***^	9.299^***^
Δ*R*^2^		0.064	0.007	0.066	0.015	0.051
Δ*F*		36.906^***^	5.096^*^	37.985^***^	9.606^**^	29.394^***^
Observations	485	485	485	485	485	485
VIF			1.03	< VIF <	1.73	

*** *p < 0.001*,

** *p < 0.01*,

* *p < 0.05*,

*+ p < 0.1*.

Similarly, compared with M9, the *R*^2^ of M8 has experienced significant increases (Δ*R*^2^ =0.015, Δ*F* = 9.606^**^), indicating that market knowledge search featured a fundamental explanation effect on disruptive innovation. Moreover, the monomial coefficient of the market knowledge search was significantly positive (β =0.318, *p* <0.001), whereas the quadratic coefficient of the market knowledge search was significantly negative (β = −0.194, *p* < 0.01). Judging from the result, the market knowledge search has an inverted U-shaped relationship with disruptive innovation, which could support H4.

Finally, compared with M5, the *R*^2^ of M10 has experienced significant increases (Δ*R*^2^ =0.051, Δ*F* = 29.394^***^), and the regression coefficient of the scientific knowledge search was significantly positive (β =0.321, *p* <0.001). Judging from the result, it could be concluded that the scientific knowledge search was positively correlated with disruptive innovation at a significant level, which could support H6.

#### Moderating Effect Test

##### Moderating Effect of the Internal Attention Focus of the Manager

To verify H7, this study has taken sustaining innovation as the dependent variable before successively introducing the control variables, independent variables, moderating variables, and interaction items between independent variables and moderating variables. As specified in Table 7, M11 is the benchmark model containing only the control variables. Subsequent to the addition of independent variables and moderating variables into the benchmark model, M12, M14, and M16 are derived, whereas the addition of interaction items between independent variables and moderating variables has led to M13, M15, and M17. Compared with M12, the *R*^2^ of M13 has shown no evident increases (Δ*R*^2^ = −0.001, Δ*F* = 0.683), whereas the interaction coefficient between the supply-chain knowledge search and the internal focus of attention was not significant (β =0.034, *p* > 0.1). Compared with M14, the *R*^2^ of M15 has not experienced significant improvements (Δ*R*^2^= −0.001, Δ*F* =0.348), whereas the interaction coefficient of the market knowledge search and the internal focus of attention was negative but not significant (β = −0.021, *p* > 0.1). Judging from the result, there was no significant correlation between interaction terms and dependent variables. Similarly, the *R*^2^ of M17 has not experienced significant improvements (Δ*R*^2^ = −0.004, Δ*F* =0.094) as opposed to M16, whereas the interaction coefficient of the scientific knowledge search and the internal focus of attention focus of the manager was not significant (β = −0.006, *p* > 0.1). Judging from the result, there was no significant correlation between interaction terms and dependent variables, and thus the result is unsupportive of H7.

**Table 7:** Moderating effect of manager's internal attention focus.

**Variable**	**Sustaining innovation**						
	**M11**	**M12**	**M13**	**M14**	**M15**	**M16**	**M17**
Constant	3.753^***^	1.332^***^	2.212+	1.566^***^	1.035	1.836^***^	1.610^*^
Company type	0.179^*^	0.094	0.09	0.101	0.1	0.108+	
Company age	0.008	−0.027	−0.029	−0.011	−0.01	0.03	0.041
Number of employees	0.106+	0.091+	0.091+	0.045	0.046	0.011	0.022
Nature of property rights	−0.135+	−0.160^*^	−0.161^*^	−0.148^*^	−0.146^*^	−0.084	−0.067
Industry	0.066	−0.068	−0.063	−0.056	−0.058	0.026	0.032
R and D investment	0.139^**^	0.150^***^	0.151^***^	0.104^*^	0.104^*^	0.066	0.065
Product strategic autonomy	0.109^*^	−0.032	−0.032	0.018	0.017	0.045	0.049
Industrial chain position	0.003	0.023	0.023	0.032	0.03	0.078	0.074
Industry attribute	−0.033	0.027	0.023	0.081	0.081	0.053	0.057
Manager's internal attention focus		0.132^***^	0.131^***^	0.123^***^	0.125^***^	0.136^***^	0.138^***^
Supply-chain knowledge search		0.468^***^	0.311				
Supply-chain knowledge search **×** Manager's internal attention focus			0.034				
Market knowledge search				0.453^***^	0.555^**^		
Market knowledge search **×** Manager's internal attention focus					−0.021		
Scientific knowledge search						0.376^***^	0.409^**^
Scientific knowledge search **×** Manager's internal attention focus							−0.006
Adj. *R*^2^	0.107	0.282	0.281	0.33	0.329	0.326	0.322
*F*	7.453^***^	18.277^***^	16.800^***^	22.641^***^	20.755^***^	22.289^***^	21.912^***^
Δ*R*^2^		0.175	−0.001	0.223	−0.001	0.219	−0.004
Δ*F*		58.821^***^	0.683	79.854^***^	0.348	78.156^***^	0.094
Observations	485	485	485	485	485	485	485
VIF			1.03	< VIF <	1.65		

*** *p < 0.001*,

** *p < 0.01*,

* *p < 0.05*,

*+ p < 0.1*.

##### Moderating Effect of the External Attention Focus of the Manager

To verify H8–H10, this study has analyzed how the focus of attention on the external situation could moderate the inverted U-shaped relationship between the supply-chain knowledge search/market knowledge search and the disruptive innovation by setting up the following models:

Dependent variable = β_0_ + β_1_ control variable + β_2_ independent variable + β_3_ independent variable^2^ + β_4_ independent variable ^*^ moderating variable + β_5_ independent variable^2^^*^ moderating variable + β_6_ moderating variable + ε; where β_0_ refers to the intercept term and ε refers to the residual item.

According to the study of Haans et al. (2016), the moderating variable can moderate the U-shaped relationship between independent variables and dependent variables by altering the position of the inflection point and the gradient of the curve. The translation direction of the inflection point on the quadratic curve is determined by the plus-minus sign of β_2_β_5_-β_3_β_4_. In addition, the moderating variable could moderate the inflection point of the U-shaped curve to the right when the β_2_β_5_-β_3_β_4_ is >0 and to the left when the β_2_β_5_-β_3_β_4_ is < 0. The plus-minus sign of β5 has determined the gradient of the quadratic curve. The moderating variable can moderate the gradient of the inverted U-shaped curve to be more gentle when the β_5_ is > 0 and to be steeper when the β_5_ is < 0.

This study has taken disruptive innovation as the dependent variable before successively introducing the control variables, independent variables, moderating variables, and interaction items between independent variables and moderating variables. As specified in Table 8, M17 is the benchmark model containing only the control variables. Subsequent to the addition of independent variables and moderating variables into the benchmark model, M18, M20, and M22 are derived accordingly. Finally, M19, M21, and M23 are generated after the addition of interaction items between independent variables and moderating variables into the benchmark model.

**Table 8:** Moderating effect of manager's external attention focus.

**Variable**	**Disruptive innovation**						
	**M17**	**M18**	**M19**	**M20**	**M21**	**M22**	**M23**
Constant	3.101^***^	1.224+	−7.909+	1.652^**^	−10.718^**^	1.080+	−3.792^*^
Company type	0.192+	0.102	0.12	0.116	0.124	0.119	0.129
Company age	0.07	0.033	0.04	0.053	0.028	0.063	0.073
Number of employees	0.031	−0.005	−0.007	−0.044	−0.037	−0.047	−0.053
Nature of property rights	−0.096	−0.132	−0.119	−0.116	−0.112	−0.076	−0.077
Industry	0.375^*^	0.123	0.168	0.152	0.134	0.25	0.24
R and D investment	0.170^*^	0.191^**^	0.188^**^	0.142^*^	0.170^*^	0.136+	0.125+
Product strategic autonomy	0.111	−0.051	−0.037	−0.012	−0.025	0.002	0.011
Industrial chain position	0.157	0.149	0.147	0.147	0.165+	0.195^*^	0.206^*^
Industry attribute	−0.482^***^	−0.415^***^	−0.387^***^	−0.391^***^	−0.371^***^	−0.426^***^	−0.405^***^
Manager's external attention focus		0.300^***^	0.333^***^	0.341^***^	0.449^***^	0.318^***^	0.295^***^
Supply-chain knowledge search		0.259^*^	1.836^**^				
Supply-chain knowledge search **×** Supply-chain knowledge search		−0.194^**^	−0.163				
Supply-chain knowledge search **×** Manager's external attention focus			−0.282^*^				
Supply-chain knowledge search **×** Supply-chain knowledge search **×** Manager's external attention focus			−0.126				
Market knowledge search				0.170^*^	2.413^***^		
Market knowledge search **×** Market knowledge search				−0.245^***^	−0.163^*^		
Market knowledge search **×** Manager's external attention focus					−0.378^***^		
Market knowledge search **×** Market -driven search **×** Manager's external attention focus					−0.332^***^		
Scientific knowledge search						0.240^***^	1.217^***^
Market knowledge search **×** Manager's external attention focus							−0.182^**^
Adj. *R*^2^	0.095	0.192	0.198	0.21	0.237	0.178	0.19
*F*	6.668^***^	10.576^***^	9.525^***^	11.71^***^	11.746^***^	10.523^***^	10.464^***^
Δ*R*^2^		0.097	0.006	0.115	0.027	0.083	0.012
Δ*F*		19.91^***^	2.752+	23.939^***^	9.445^***^	24.856^***^	8.086^***^
Observations	485	485	485	485	485	485	485
VIF			1.03	< VIF <	3.31		

*** *p < 0.001*,

** *p < 0.01*,

* *p < 0.05*,

*+ p < 0.1*.

Compared with M18, the *R*^2^ of M19 has experienced significant increases (Δ*R*^2^ =0.006, Δ*F* = 2.752+), whereas the regression coefficient of the interaction term between the monomial of the supply-chain knowledge search and the external focus of attention was significantly negative (β = −0.282, *p* <0.05). However, the regression coefficient of the interaction term between the quadratic term of the supply-chain knowledge search and the external focus of attention was negative but not significant (β = −0.282, *p* <0.05). Subsequent to the addition of the monomial, quadratic term and the interaction term of the supply-chain knowledge search and the external focus of attention of the manager into the regression equation, β_2_ = 1.836, β_3_ = −0.163, β_4_ = −0.282, β_5_ = −0.126, β_2_β_5_-β_3_β_4_ = −0.277302 < 0. Thus, the results supported H8.

Compared with M20, the *R*^2^ of M21 has experienced significant improvements (Δ*R*^2^ =0.027, Δ*F* = 9.445^***^), whereas the interaction coefficient of the monomial of the market knowledge search and the external focus of attention was negative and significant (β = −0.378, *p* < 0.001), as well as the interaction coefficient of the quadratic term of the market knowledge search and the external focus of attention (β = −0.332, *p* < 0.001). Based on M21, in the regression equation, β_2_ = 2.413, β_3_ = −0.163, β_4_ = −0.378, β_5_ = −0.332, β_2_β_5_-β_3_β_4_ = −0.86273 < 0. Thus, the external focus of attention would cause the inflection point of the inverted U-shaped curve between the market knowledge search and the disruptive innovation to move to the left or to become steeper. Hence, the result could support H9.

Compared with M22, the *R*^2^ of M23 has experienced significant improvements (Δ*R*^2^ =0.012, Δ*F* = 8.086^***^), whereas the interaction coefficient of the scientific knowledge search and the external focus of attention was significantly negative (β = −0.182, *p* < 0.01). Judging from the result, there was a significant negative correlation between the interaction terms of the scientific knowledge search and the external focus of attention and the dependent variables, which could support H10.

## Conclusions and Discussion

According to the knowledge-based theory and the open innovation theory, the model of conceptual relationship was established in this study through the theoretical-deductive approach, to elaborate on the links between the specialized knowledge search, the focus of attention of the manager, and the sustaining innovation, as well as the disruptive innovation. Through the in-depth study into the influencing mechanism of the specialized knowledge search on the sustaining innovation and disruptive innovation, we reached the conclusions specified as follows:

The specialized knowledge search could impose a profound and positive impact on corporate innovation. However, the importance of external partners to corporate innovation could vary in accordance with their categories. Specifically, sustaining innovation was positively correlated with the three strategies of knowledge search, namely, the supply-chain knowledge search, the market knowledge search, and the scientific knowledge search. Moreover, the disruptive innovation was found to have an inverted U-shaped relationship with the supply-chain knowledge search and the market knowledge search, while featuring a positive correlation with the scientific knowledge search. The supply-chain knowledge search provided the strongest momentum for sustaining innovation, followed by the market knowledge search, whereas the scientific knowledge search has imposed the slightest effect on facilitating the sustaining innovation. This research finding reaffirmed the importance of the external knowledge search and clarified the external knowledge sources related to corporate innovation, indicating that manufacturing enterprises ought to strategically adjust the scope and object of their knowledge search.

The focus of attention of the manager could play a vital role in the interaction between the knowledge search and corporate innovation. Specifically, judging from the research findings, the moderating effect of the internal focus of attention of the manager was not significant on the three types of knowledge search and sustaining innovation. On the one hand, when managers paid more attention to the internal environment, they were able to ensure that their enterprises could perceive the changes taken place in the market more quickly, and then transform the knowledge searched from external entities into new products and services to cope with technological changes and innovation. Such transformation could help reflect the value of new technology or knowledge and would enable enterprises to maintain and improve their competitiveness. However, on the other hand, the sustaining innovation highlighted the need for enterprises to bring external factors (such as external environment, policy, and market) into the scope of decision-making information, thereby construing the competitive pressure faced by enterprises and the changes to the demands of customers at present. In case managers pay excessive attention to merely internal factors, they risk isolating the exchanges of external information (Li et al., 2013), which may lead to the failure of their enterprises to promptly respond to the changes of customer demands and technological progress at present. This may weaken the impact imposed by the internal focus of attention on corporate innovation, and in this case, it is hard to strengthen the positive impact of the specialized knowledge search on sustaining innovation.

Moreover, the results of this study indicated that the external focus of attention could negatively moderate the relationship between disruptive innovation and the three methods of knowledge search. According to Khanagha et al. (2013), the knowledge obtained by enterprises through the external knowledge search is prone to produce internal resistance, leading to the not-invented-here (NIH) syndrome of the organization, i.e., the “not invested here” syndrome. In other words, when enterprises are acquiring knowledge from their competitors, suppliers, customers, universities, scientific research institutes, and other external entities, the external focus of attention would render the procedure of knowledge identification and evaluation irrational under the authorization of managers. Under such circumstances, it would be harder for the knowledge of the competitor to play its due role. Therefore, the external focus of attention may strengthen the inhibitory effect imposed by the knowledge search on the disruptive innovation, weaken the promoting effect, and ultimately have an utterly negative impact. The moderating effect of the focus of attention of the manager in adjusting the relationship between the specialized knowledge search and the corporate innovation could fully reflect the difference of its influencing mechanism.

In this study, an in-depth analysis was carried out on the relationship between the specialized knowledge search and the corporate innovation performance, whereas the critical role of external knowledge sources on the corporate innovation performance was further clarified. Judging from the previous studies on the relationship between the external knowledge search and the innovation performance, scholars did not reach a consensus on their specific connection. Most of the existing studies have divided the dimensions of the external knowledge search based on their respective research purposes. Even during the studies on the search width and the search depth, scholars are still attempting to elaborate on their impact on the innovation performance, and there has been no unified opinion on the issue. This study has taken a holistic look into the role of varying types of knowledge search in sustaining innovation and disruptive innovation. It is expected to enrich the existing studies on the influencing factors of sustaining innovation and disruptive innovation while providing theoretical and practical guidance for enterprises to better facilitate innovation through the knowledge search. The research findings did not only lay an empirical foundation for the related theories of predecessors but also provided a reference value for the follow-up in-depth studies of other scholars to some extent.

As evidenced by previous studies, the knowledge search could facilitate the corporate innovation behavior, but these studies have neglected the effect imposed by the cognitive features of the managers on the innovation process. It was found that the cognitive model of the manager could determine their interpretation of the knowledge search. This study has taken the focus of attention of the manager as a moderating variable into the theoretical model to elaborate on the differences between the internal focus of attention and the external one along the impact path of the specialized knowledge search on the sustaining innovation and disruptive innovation. Furthermore, the study has clarified the specific procedure of the influence of the senior manager in facilitating corporate innovation. In addition, the study could shed light on the boundary conditions of the impact imposed by the specialized knowledge search on sustaining innovation and disruptive innovation.

## Data Availability Statement

The raw data supporting the conclusions of this article will be made available by the authors, without undue reservation.

## Ethics Statement

This research was carried out in accordance with the recommendations of moral rule for empirical research, and approved by the Academic Committee of Business School of Huaqiao University; meanwhile all the survey respondents were given written informed consent.

## Author Contributions

XL and ZY contributed to the ideas of the research, collection of data, and empirical analysis. CL, ZY, and DZ contributed to the data analysis, design of research methods, and tables. CL, XL, and ZY participated in developing a research design, writing, and interpreting the analysis. All authors participated in reading and approval of the final manuscript. All authors have read and agreed to the published version of the manuscript.

## Funding

This research project was supported by the Scientific Research Initiation Project for High-level Talents of Huaqiao University (Grant No. 15SKBS204) and the National Natural Science Foundation of China (Grant No. 71974059).

## Conflict of Interest

The authors declare that the research was conducted in the absence of any commercial or financial relationships that could be construed as a potential conflict of interest.

## Publisher's Note

All claims expressed in this article are solely those of the authors and do not necessarily represent those of their affiliated organizations, or those of the publisher, the editors and the reviewers. Any product that may be evaluated in this article, or claim that may be made by its manufacturer, is not guaranteed or endorsed by the publisher.
