# Supplementary material for: The Impact of Specialized Knowledge Search on Enterprise Innovation
Source: Front Psychol. 2021 Oct 7;12:725514. doi: 10.3389/fpsyg.2021.725514 (PMC8529040; doi:10.3389/fpsyg.2021.725514)
Supplement: Supplementary file 1 [file Presentation_1.pdf]

Appendix A. Questionnaire Items of The Study

Sample characteristics

1: Founding years of your company

□less than 5 years; □less than 5-10 years; □less than 10-15 years; □more than 15 years;

2: Number of employees

□less than 20 people; □less than 20-300 people; □less than 300-1000 people; □more than 1000 people

3: Type of your company

□headquarters of the group company; □subsidiaries of the group company; □branches of the group company; □independent legal person enterprise not affected by other companies; □Business Department of the group company; □others __.

4: The nature of the property

□state owned enterprises; □private enterprises; □foreign funded enterprise; □Sino foreign joint venture;

5: Compared with major competitors, the R & D Investment of the enterprise

□strongly lower; □lower; □general; □higher; □strongly higher

6: Supply chain position of the enterprise

□upstream; □midstream; □downstream

7: Nature of industry

□strategic emerging industry; □traditional industries

Christensen defined disruptive innovation as innovation in which an enterprise was based on a low-end niche market and increased its market share by meeting customers' needs, thus undermining the position of the incumbent enterprise.

I. Based on the definition of disruptive innovation, indicate your level of agreement with the following statements, "1" meaning "strongly disagree" and "7" meaning "strongly agree"

Items	Options
Q1: The new products developed by the enterprise could present utterly strong features of disruptive innovation	1 2 3 4 5 6 7
Q2: Companies often develop new products with the nature of disruptive innovation	1 2 3 4 5 6 7
Q3:The enterprise could play a leading role in developing the disruptive products	1 2 3 4 5 6 7
Q4: New products developed by the company are very attractive to non-mainstream	1 2 3 4 5 6 7

customers or customers in specific markets	
Q5: New products developed by the enterprise are utterly attractive to the customers inclined towards anti-establishment and the market-specific customers	1 2 3 4 5 6 7

Sustaining innovation aims to meet the needs of mainstream users in the existing market and promote the performance and quality of existing products to become better and higher through continuous improvement.

II. Based on the definition of sustaining innovation, indicate your level of agreement with the following statements, "1" meaning "strongly disagree" and "7" meaning "strongly agree"

**Items**	**Options**
Q6: In the past five years, most of the innovation activities carried out by the company focused on the performance improvement of the original products and services, so as to better meet the needs of customers in the mainstream market	1 2 3 4 5 6 7
Q7: The business entities are the pioneers among peers in terms of constantly improving their products in the mainstream market,	1 2 3 4 5 6 7
Q8: The innovation activities in the business entities could satisfy and continue to draw interest from the mainstream market or high-end market.	1 2 3 4 5 6 7
Q9: The innovation activities carried out by your company attract the mainstream customer market	1 2 3 4 5 6 7
Q10: The innovation activities carried out by your company pay little attention to the needs of non- mainstream markets.	1 2 3 4 5 6 7

III. Indicate your level of agreement with the following statements, "1" meaning "strongly disagree" and "7" meaning "strongly agree"

**Items**	**Options**
Q11: We often obtain innovation knowledge and information from suppliers	1 2 3 4 5 6 7
Q12: We regularly collect customers' opinions and suggestions on the management of the company	1 2 3 4 5 6 7
Q13: We often obtain innovation knowledge and information from industry associations	1 2 3 4 5 6 7
Q14: We often search for innovation knowledge and information from research	1 2 3 4 5 6 7

institutions	
Q15: We often search for innovation knowledge and information through trade fairs	1 2 3 4 5 6 7
Q16: We often obtain innovation knowledge and information from competitors	1 2 3 4 5 6 7
Q17: We often communicate with enterprises in other industries to obtain innovation knowledge and information	1 2 3 4 5 6 7
Q18: We often search for innovation knowledge and information from management consulting institutions	1 2 3 4 5 6 7
Q19: We often search knowledge and information about innovation from research institutions in the other sectors	1 2 3 4 5 6 7
Q20: We often cooperate with colleges and universities to search for innovation knowledge and information	1 2 3 4 5 6 7
Q21: We often cooperate with scientific research institutes to search for innovation knowledge and information	1 2 3 4 5 6 7
Q22: We often obtain knowledge and information on innovation through professional meetings or forums	1 2 3 4 5 6 7
Q23: We often search for innovation knowledge and information from relevant government departments	1 2 3 4 5 6 7
Q24: We often search for innovation knowledge and information from private research institutions	1 2 3 4 5 6 7

IV. Indicate your level of agreement with the following statements, "1" meaning "strongly disagree" and "7" meaning "strongly agree"

**Items**	**Options**
Q25: CEO pays much attention to the issues of enterprise organization, and	1 2 3 4 5 6 7
Q26: CEO pays great attention to the problems of employees	1 2 3 4 5 6 7
Q27: CEO attached great importance to the interests of corporate shareholders	1 2 3 4 5 6 7
Q28: CEO pays much attention to the demands of customers	1 2 3 4 5 6 7
Q29: CEO pays great attention to changes in customers' purchasing power	1 2 3 4 5 6 7
Q30: CEO pays much attention to the changes taken place in the industry.	1 2 3 4 5 6 7
